# Diarrhea, Stimulation and Growth Predict Neurodevelopment in Young North Indian Children

**DOI:** 10.1371/journal.pone.0121743

**Published:** 2015-03-31

**Authors:** Ingrid Kvestad, Sunita Taneja, Mari Hysing, Tivendra Kumar, Nita Bhandari, Tor A. Strand

**Affiliations:** 1 Department of Biological and Medical Psychology, Faculty of Psychology, University of Bergen, Bergen, Norway; 2 Centre for Child and Youth Mental Health and Child Welfare, Uni Research Health, Bergen, Norway; 3 Society for Applied studies, New Delhi, India; 4 Society for Essential Health Action and Training, New Delhi, India; 5 Centre for International Health, University of Bergen, Bergen, Norway; 6 Department of Laboratory Medicine, Innlandet Hospital Trust, Lillehammer, Norway; TNO, NETHERLANDS

## Abstract

**Background and Objective:**

Infants and young children in low to middle-income countries are at risk for adverse neurodevelopment due to multiple risk factors. In this study, we sought to identify stimulation and learning opportunities, growth, and burden of respiratory infections and diarrhea as predictors for neurodevelopment.

**Methods:**

We visited 422 North Indian children 6 to 30 months old weekly for six months. Childhood illnesses were assessed biweekly. At end study, we assessed neurodevelopment using the Ages and Stages Questionnaire 3^rd^ ed. (ASQ-3) and gathered information on stimulation and learning opportunities. We identified predictors for ASQ-3 scores in multiple linear and logistic regression models.

**Results:**

We were able to explain 30.5% of the variation in the total ASQ-3 score by the identified predictors. When adjusting for child characteristics and annual family income, stimulation and learning opportunities explained most of the variation by 25.1%. Height for age (standardized beta: 0.12, p<.05) and weight for height z-scores (std. beta: 0.09, p<.05) were positively associated with the total ASQ-3 score, while number of days with diarrhea was negatively associated with these scores (std. beta: -0.13, p<0.01).

**Conclusion:**

Our results support the importance of early child stimulation and general nutrition for child development. Our study also suggests that diarrhea is an additional risk factor for adverse neurodevelopment in vulnerable children.

## Introduction

There is sound evidence that deficient care and inadequate stimulation are key risk factors for adverse neurodevelopment in children [1–3]. Likewise, the evidence for poor growth and stunting as significant risk factors is convincing [4,5]. Pneumonia and diarrhea are important causes of morbidity and mortality in children in low to middle-income countries (LMIC) [6]. In some studies, diarrhea prevalence has predicted neurodevelopment [4,7–9], but a recent meta-analysis including these studies concluded that number of days with diarrhea did not predict neurodevelopment when taking stunting into account [10]. However, the very few studies included in this meta-analysis varied substantially in sample size, age of the participants, choice of cognitive measures and the quality of data on diarrhea.

In poor populations, risk factors co-occur giving rise to cumulative effects on neurodevelopment [2,5,11,12]. Complex relationships among these risk factors make it challenging to determine their independent contribution. In the present study, we have assessed developmental status and collected information on various risk and protective factors for adverse development such as socioeconomic status, child characteristics and stimulation and learning opportunities in a sample of 422 young North Indian children. The children participated in a six months study of folic acid and vitamin B12 supplementation on growth, diarrhea and other infections in New Delhi, India [13], and unique to this study is the thorough biweekly assessment of childhood illnesses such as acute lower respiratory infections (ALRI), pneumonia and diarrhea. The main aim of our study is to identify predictors for neurodevelopment in multiple regression models, and specifically to measure the extent to which diarrheal illness is associated with early child development.

## Materials and Methods

### Participants and study setting

The children (n = 422) included in this study were part of a randomized, doubled blind, placebo controlled trial (RCT) (n = 1000) on the effect of vitamin B12 and/or folic acid supplementation on childhood infections and growth in New Delhi, India (clinicaltrials.gov: NCT 00717730) [13]. Children aged 6 to 30 months were enrolled and randomized in blocks of 16, the last 440 randomized enrollments were requested to participate in the developmental assessment sub study. Of these, three children were not available for assessment and 15 did not wish to participate, hence the final number of participants was 422. The enrollment for this sample was from November 2010 through March 2011, and the developmental assessments were performed from May through September 2011. The study site was in the low and middle socioeconomic settings of Tigri and Dakshinpuri in New Delhi. These are typical urban neighborhoods with a total population of about 300,000. The ethics committees of the Society for Essential Health Action and Training (India), Society for Applied Studies (India), Christian Medical College (India), and the Norwegian Regional Committee for Medical and Health Research Ethics (REK VEST) approved the study.

### Procedure

For enrollment, a door-to-door survey was conducted to identify households with eligible children. A physician and field supervisors screened the children and written informed consent was obtained from caregivers prior to enrollment. Availability of informed consent and no plans to move away over the next 6 months were considered for enrollment. We excluded children with severe acute malnutrition (weight for height z-scores less than -3), with severe anemia (hemoglobin<7 g/dL), and those who were using folic acid and/or vitamin B12 supplements. Information on child characteristics and socioeconomic status was collected at baseline. A team of field workers visited the children`s household twice weekly for six months for close morbidity follow up. Weight and height was measured at baseline and at end study at the study clinic. At the end of six months follow up, developmental assessment was conducted and information on the child`s stimulation and learning opportunities was collected at the study clinic. There were no additional exclusion criteria for the developmental assessment.

### Measurements

#### Developmental assessment

Neurodevelopmental status was measured by the Ages and Stages Questionnaire 3. ed. (ASQ-3), a comprehensive checklist, standardized for children 1–66 months with age-appropriate questionnaires [14]. The questionnaires contain 30 items that sums up to five subscales: Communication, Gross motor, Fine motor, Problem-solving and Personal-social (possible score range from 0 to 60), and a total score (possible score range form 0 to 300). Three field supervisors were trained to administer the ASQ-3 directly with the child at the research clinic in the presence of caregivers. The examiners elicited the relevant skills from the child during sessions using standardized materials. The caregiver served as an important contributor in supporting the child, eliciting behaviors and gave relevant information of the child’s development when necessary. The three field supervisors were trained by the main author, a clinical child psychologist with experience in the assessment of infants and young children and in training of personnel.

All forms were translated to Hindi following official recommendations [15], and items not appropriate for the cultural setting were identified and slightly adjusted (for extensive information see [16]). During the 11 days of training, the field supervisors were standardized in performing the procedure, and they reached a high inter-observer agreement both during training and in the 10% quality control throughout the study. In the translated ASQ-3 version, the standardized alphas for the total ASQ-3 scores were strong, indicating an overall acceptable internal consistency [16].

#### Stimulation and learning opportunities

To assess the caregiver`s promotion of child development we carefully selected two questions from the standardized assessment tool the Home Observation for Measurement of the Environment (HOME) [17]. One question was on “Mother`s belief that child`s behavior can be modified” and one was on “Mother`s encouragement of developmental advances”. These questions and other questions on the child`s stimulation and learning environment, such as number of toys and books in the home, hours of play with other children and attendance to anganwadi centre (childcare) were asked the caregivers during the sessions.

#### Childhood illnesses and growth

At the biweekly field worker visits, mothers were asked about diarrheal illness, symptoms of respiratory infections and fever on any day since the last visit, and whether treatment had been sought for any illness. Respiratory rates were counted twice at each visit, temperature was measured and the child was examined for signs of dehydration if diarrhea or vomiting were present. Diarrhea was defined as the passage of ≥3 loose or watery stools in a 24-h period. ALRI was defined as cough or difficult breathing with elevated respiratory rate above the age-specific cut-off values (≥50 breaths per min in infants and ≥40 breaths per min in older children) according to WHO-criteria, or cough or difficult breathing and lower chest indrawings. Clinical pneumonia was defined either by a combination of cough with crepitations or bronchial breathing by auscultations or as an episode of acute lower respiratory tract infection associated with at least one of the following features; lower chest indrawings, convulsions, not able to drink or feed, extreme lethargy, restlessness or irritability, nasal flaring or child is abnormally sleepy and difficult to wake up.

Anthropometry was assessed through weight and length measurements at baseline and end study at the study clinic. Weight was measured to the nearest 50 g using Digitron scales. Height was measured using locally manufactured infantometers reading to the nearest 0.1 cm.

### Data management and statistical analyses

The data was double entered by two data entry operators followed by validation. A total of 0.21% of the ASQ-3 responses were missing. For missing items an adjusted total score was computed by dividing the total subscale score by the number of completed items [18]. This number was then added depending on the amount of items missing. For each child, we summed up the item scores to five total subscale scores, and a total ASQ-3 score. We measured the association of relevant independent variables with the total ASQ-3 in multiple linear regression models. We selected the variables for the regression models as described elsewhere [19]. The variables that were included in the initial crude models were: number of family members, mother’s age, mother’s year of schooling, father’s year of schooling, if family owns television or scooter or cooler, annual family income, joint versus nuclear family, attendance in anganwadi, number of toys in the family, family owns books, number of children in the family, hours of play with other children during the week, mothers belief that child`s behavior can be modified, mothers encouragement of developmental advances, height for age z-scores (HAZ), weight for height z-scores (WHZ), number of days with diarrhea, incidents of clinical pneumonia and incidents of ALRI. Due to collinearity, weight for age z-scores were not included in the adjusted models. We confirmed this manual model by selecting variables in an automatic stepwise linear regression procedure. For the regression models the log-transformed values of annual family income and the log(base2) transformed values of days of diarrhea were used.

For the total ASQ-3 score, we present the selected variables in groups using a hierarchical (nested) regression approach [20]. The variable groups are: stimulation and learning opportunities (number of toys in the family, family owns books, hours of play with children during the week, mothers belief that child`s behavior can be modified, mothers encouragement of developmental advances), growth (HAZ and WHZ) and childhood illnesses (number of days with diarrhea and incidence of clinical pneumonia). The variable groups were entered in the analysis in different steps constituting different models. For instance, stimulation and learning opportunities was entered in step 1 constituting model 1. In model 4 growth variables were added, and in model 7 the childhood illnesses variables were added to a full model. The remaining models (2, 3, 5 and 6) constitute different constellations of the variable groups alone and together. All regression models were adjusted for child characteristics (sex, age and breastfeeding status) and annual family income. The child characteristics variables were included regardless of their significance or influence on the other variables in the initial crude models.

The scores of the five subscales were highly skewed and categorized on the 25^th^ percentile in the multiple logisitic regression analysis. The selection of variables followed the same procedure as for main regression analysis. Only variables with P>0.05 are presented in the table. Data was analyzed in Stata version 12.

## Results

Of the 440 children three children were not available for assessment and 15 refused to participate. The final number of participants was 422.

### Demographic Characteristics

Demographic information of the children in the cohort is shown in Table 1. There was an even distribution of girls and boys. Most of the children were breastfed (86.3% at baseline), 40.1% were stunted (<-2 HAZ), 10% were wasted (<-2 WHZ) and 31% were underweight (<-2 WAZ). The average days of diarrhea during the study period, were 6.6 days (SD: 7.1), 14% of the children had no episodes of diarrhea, 53.3% had between 1–7 days and 32.7% had between 8–49 days with diarrhea during the 6 months period. At least one episode of ALRI was reported in 37.7%, and clinical pneumonia in 27.2% throughout the observation period.

**Table 1:** Demographic and clinical characteristics of children in the cohort.

**Baseline**				N	Mean/%	SD
**Child characteristics**						
	Total			422		
	Age in month					
		12–23 months		259	61.3%	
		24–36 months		163	38.7%	
	Sex					
		Girls		206	48.8%	
	Breastfed			364	86.3%	
**Family situation**						
	***Economy***					
		Annual income in INR^1^ (median/range)			73000	12000–870000
		Families who own color TV or scooter or cooler, n (%)		377	89.3%	
	***Maternal characteristics***					
		Age			25.7	5.5
		Years of schooling			7	6.3
		Mother’s occupation, n (%)^2^				
			Governmental employee	1	0.2%	
			Non-governmental employee	8	1.9%	
			Self employed	7	1.7%	
			Daily wager, maid or un-employed	405	96%	
	***Paternal characteristics***					
		Years of schooling			8.6	4
		Father’s occupation, n (%)				
			Governmental employee	9	2.1%	
			Non-governmental employee	237	56.2%	
			Self employed	89	21.1%	
			Daily wager or un-employed	87	20.6%	
	***Household characteristics***					
		Type of family				
			Nuclear, n (%)	228	54%	
			Joint, n (%)	194	46%	
		Number of children in the family			3	2.3
		Family size			5.8	2.6
**Stimulation and learning opportunities**						
	Hours of weekly play with other children				19	16.6
	Number of toys in the family					
		No toys		16	3.8%	
		Less than 5 toys		120	28.4%	
		5–10 toys		147	34.8%	
		More than 10 toys		139	32.9%	
	Families who owns books			253	60%	
	Attending Anganwadi center^3^, n (%)			40	9.5%	
**Anthropometry**						
	Z score height for age (stunted), < -2, n (%)			169	40.1%	
	Z score weight for length (wasted), <- 2, n (%)			42	10%	
	Z score weight for age (underweight), < -2, n (%)			131	31%	
**Childhood Illnesses from the biweekly home visits throughout the study period**						
	Number of days with diarrhea				6.6 days	7.1
	Incidents of Acute lower respiratory infection			159	37.7%	
	Incidents of Clinical Pneumonia			115	27.2%	

^*1*^ *Indian Rupees*,

^*2*^ *One mother is deceased*,

^*3*^ *Childcare center*

### Predictors for developmental status

The predictors for the total ASQ-3 scores are shown in Table 2. All models were adjusted for child characteristics and annual family income. These variables explained 4.7% of the variation in the total ASQ-3 score alone. In the full model (model 7), all variables together explained 30.5% of the variation.

**Table 2:** Hierarchical Regression Analysis for variables predicting total ASQ-3 scores in North Indian children 12–36 months^1^.

		**Model 1**	**Model 2**	**Model 3**		**Model 4**	**Model 5**	**Model 6**		**Model 7**	
*Adjusted for Child Characteristics and annual family income* ^*2*^											
**VARIABLES**		B^*3*^ (SE)	B (SE)		B (SE)	B (SE)	B (SE)	B (SE)	B (SE)		β^*4*^
**Stimulation and learning opportunities**											
Number of toys											
	More than 10 toys	ref.				ref.	ref.		ref.		
	6–10 toys in the home	-4.4 (5.4)				-2.9 (5.3)	-2.2 (5.4)		-2.4 (5.2)		
	1–5 toys in the home	-5.8 (5.8)				-4.0 (5.8)	-2.8 (5.9)		-3.4 (5.7)		
	No toys in the home	-68.5***(12.0)				-59.9*** (12.0)	-61.6*** (12.1)		-57.9*** (11.9)		-0.21
Family own books (ref. No books)		4.9 (4.5)				5.7 (4.4)	5.7 (4.5)		7.0 (4.4)		
Hours of weekly play with other children		0.6*** (0.1)				0.6*** (0.1)	0.6*** (0.1)		0.6*** (0.1)		0.20
Mother`s belief that child`s behavior can be modified (ref. No modification)		14.2** (5.0)				13.2** (4.9)	12.4*(4.9)		11.7* (4.9)		-0.11
Mother`s encouragement of developmental advances (ref. No encouragement)		18.0** (5.4)				17.7** (5.3)	17.0** (5.4)		17.2** (5.3)		-0.16
**Growth**											
Height for age z-scores			7.8*** (2.2)			5.6* (2.0)		7.5** (2.1)	5.4* (2.0)		0.12
Weight for height z-scores			7.4** (2.6)			5.4* (2.4)		6.9** (2.6)	5.0* (2.4)		0.09
**Childhood Illnesses**											
Number of days with Diarrhea					-5.7** (1.8)		-5.1** (1.6)	-5.2** (1.8)	-5.0** (1.6)		-0.13
Incidents of Clinical Pneumonia					-12.6* (5.3)		-8.9 (4.9)	-12.1* (5.2)	-9.4 (4.8)		-0.08
Observations		421	421		421	421	421	421	421		
**R-squared**		**0.251**	**0.104**		**0.086**	**0.279**	**0.284**	**0.137**	**0.305**		

*** p<.001,

** p<.01,

* p<.05,

^1^ For the 422 assessed children, the mean total ASQ-3 score was 231.9 (SD = 50) with scores ranging from a minimum of 30 to a maximum of 300.

^*2*^ All models are adjusted for child characteristics (age, sex and breastfeeding status), and annual family income,

^*3*^ unstandardized Beta coefficient,

^*4*^ standardized regression coefficient, Beta values for model 7 only.

#### Stimulation and learning opportunities

Stimulation and learning opportunities adjusted for child characteristics and annual family income, explained most of the variation in the total ASQ-3 scores alone by 25.1% (Table 2, model 1). When growth was added (Table 2, model 4), 27.9% of the variation was explained, while 28.4% was explained in the model including stimulation and learning opportunities and childhood illnesses (Table 2, model 5). Four variables of the stimulation and learning opportunities were significantly associated with the ASQ-3 score. Compared to those who had more than ten toys, those who had no toys in the home had substantially lower ASQ-3 scores (p<0.001 in all models). Number of hours of weekly play with other children (p<0.001 in all models), mother`s belief that child`s behavior can be modified (p<0.01 and p<0.05) and mother`s encouragement of developmental advances (p<0.01 in all models) were all positively and significantly associated with the total ASQ-3 score.

#### Growth

**Fig 1:**
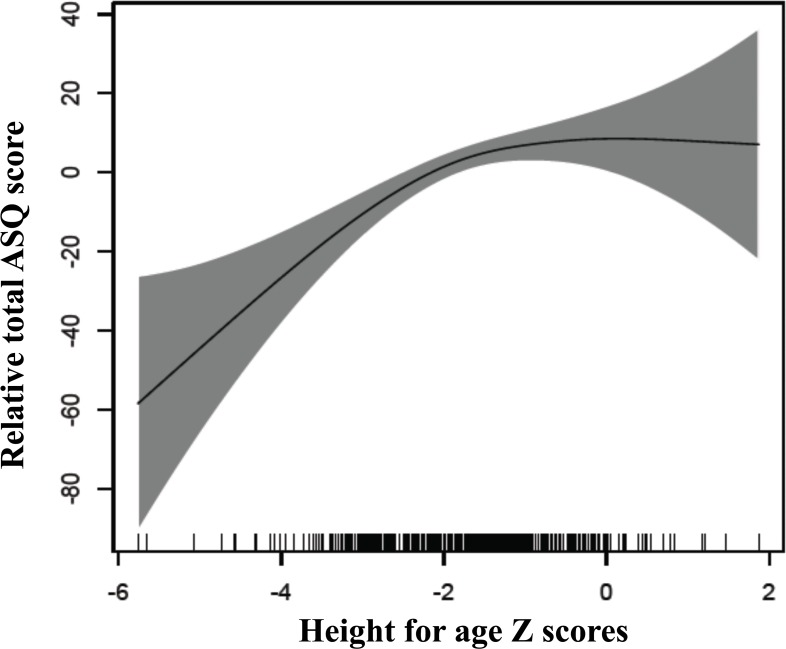
Associations between height for age z-scores and changes in ASQ-scores. The graphs were constructed using generalized additive models in R, the solid line depicts the association of the ASQ-score and HAZ. The Y-axis is centered on the mean total ASQ-score. The shaded area spans the 95% confidence interval of this association.

Growth alone explained 10.4% of the variation in the total ASQ-3 score (Table 2, model 2), and when including childhood illnesses these explained 13.7% together (Table 2, model 6). HAZ and WHZ were positively and significantly associated with the total ASQ-3 score in all models (p<0.001, p<0.01, p<0.05), however the coefficients were attenuated when stimulation and learning opportunities was included. Fig. 1 shows the relationship between HAZ and the total ASQ-3 score in generalized additive models (GAM).

#### Childhood illnesses

**Fig 2:**
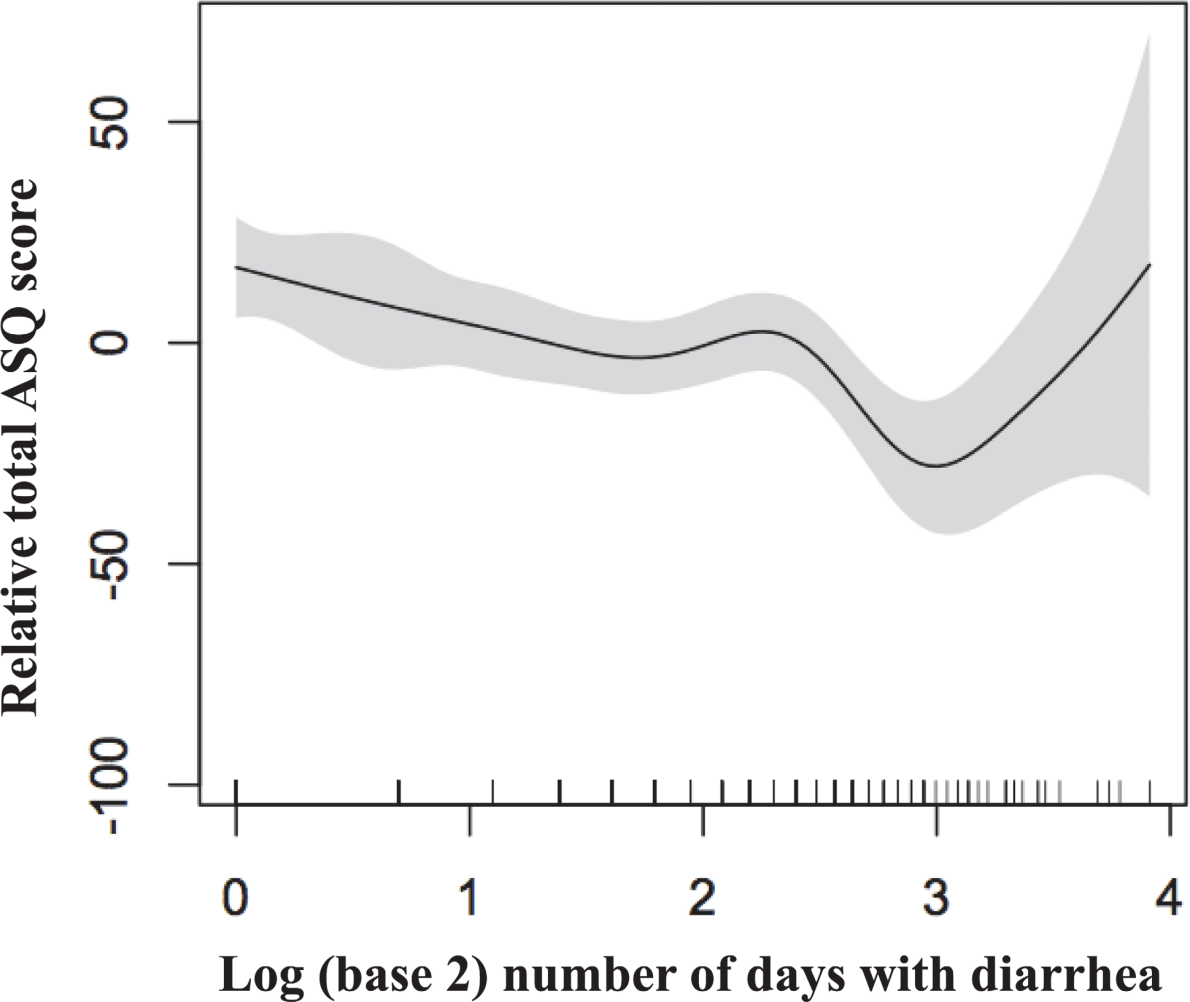
Associations between log (base2) days of diarrhea and changes in ASQ-scores. The graphs were constructed using generalized additive models in R, the solid line depicts the association of the total ASQ-score and log (base2) days of diarrhea. The Y-axis is centered on the mean total ASQ-score. The shaded area spans the 95% confidence interval of this association.

The adjusted analysis of the childhood illnesses variables explained 8.6% of the variation in the total ASQ-3 score alone (Table 2, model 3). Number of days with diarrhea was negatively and significantly associated with the total ASQ-3 score (p<0.01 in all models). Fig. 2 shows the relationship between number of days with diarrhea and the total ASQ-3 score. Clinical pneumonia was significantly associated with the total ASQ-3 score in models where stimulation and learning opportunities was not present (p<0.05 in both models) (Table 2, model 3 and 6).

#### Variables predicting the ASQ-3 subscales

Table 3 shows the predictors for the ASQ-3 subscale scores from logistic regression models. Number of days with diarrhea was significantly associated with the Fine motor and Problem- solving subscales, and incidents of pneumonia with Communication and Fine motor subscales. HAZ was significantly associated with the Gross motor subscale only, while WHZ was significantly associated with the Communication subscale.

**Table 3:** Variables predicting ASQ-3 subscale score in North Indian children 12–36 months^1^.

			**Communication**	**Gross Motor**	**Fine Motor**	**Problem Solving**		**Personal Social**
**VARIABLES**			Odds Ratio (CI)	Odds Ratio (CI)	Odds Ratio (CI)	Odds Ratio (CI)		Odds Ratio (CI)
**Child Characteristics and Socioeconomics status**								
	Age in months		0.95** (0.92–1.00)					1.04** (1.01–1.08)
	Mothers years of schooling			0.92** (0.88–0.97)				
	Annual family income						0.63* (0.44–0.90)	0.69* (0.50–0.96)
**Stimulation and learning opportunities**								
	Number of toys in the home							
		More than 10 toys in the home	ref.	ref.	ref.		ref.	ref.
		6–10 toys in the home						
		1–5 toys in the home	1.91* (1.04–3.50)					
		No toys in the home	11.04** (2.69–45.29)	3.88* (1.08–13.91)			4.31* (1.34–21.79)	
	Family own books (ref. No books)							
	Hours of weekly play with other children		0.98* (0.97–1.00)		0.98*** (0.96–0.99)		0.97*** (1.90–4.86)	0.98** (0.97–0.99)
	Mother`s belief that child`s behavior can be modified (ref. No modification)				0.34*** (0.52–0.22)			
	Mother’s encouragement of developmental advances (ref. No encouragement)		0.58* (0.53–0.21)				0.33*** (0.53–0.21)	0.47** (0.99–0.93)
**Growth**								
	Height for age z-scores			0.69 ***(0.56–0.84)				
	Weight for height z-scores		0.67** (0.51–0.86)					
**Childhood Illnesses**								
	Number of days with diarrhea				1.26** (1.07–1.49)		1.19* (1.01–1.40)	
	Incidents of Clinical Pneumonia		1.85* (1.13–3.03)		1.63* (1.00–2.62)			

*** p<.001,

** p<.01,

* p<.05, logistic regression P-value,

^1^ For the 422 assessed children, the mean subscales scores vary from 44.8 to 47.8, all with a range from 0 to 60.

## Discussion

We were able to explain 30.6% of the variation in the total ASQ-3 score by the included predictors for neurodevelopment. Stimulation and learning opportunities was the variable group that explained most of the variation. Growth was also independently associated with developmental status. Furthermore, the variable days of diarrhea was an independent and consistent predictor for the ASQ-3 scores.

Factors in children`s home environment, such as responsive caregiving and early learning opportunities are of indisputable importance for child development [2,21]. In our results this is clearly demonstrated by the variables on stimulation and learning opportunities explaining most of the variability of the neurodevelopmental scores alone. Stunting is another well-established risk factor for adverse neurodevelopmental outcomes [12]. This is supported in our study by the linear relationship between the ASQ-3 scores and HAZ-scores below -2, where the total ASQ-3 scores increase with increasing HAZ scores (Fig. 1). Furthermore, HAZ was associated with the total ASQ-3 scores with effect sizes ranging from 5.1 to 6.7 ASQ-3 points in all models. The effects of growth were seemingly stronger in models where stimulation and learning opportunities were not included.

Each doubling of the number of days with diarrhea was associated with an average decrement of approximately five ASQ-3 points. The plots from the GAM revealed that this relation was linear (Fig. 2). Our results support previous findings, for example from a prospective cohort study in Brazil, reporting of associations between early childhood diarrhea and various developmental domains in later childhood [7–9]. These reports have been criticized, however, for not adequately adjusting for environmental and health related factors, as well as for their low sample size. Furthermore, it has been argued that stunting is a relevant cofounder in the association between diarrhea and cognitive development and that diarrhea morbidity only has an effect on the developing brain through stunting [4,6,10]. By demonstrating the significant association between diarrhea and neurodevelopment independent of growth, the present study improved upon previous findings. The assessment of illnesses was conducted biweekly for six months and we have information on several potential confounders, the results are thus based on a more extensive assessment then previous studies.

Analysis on the separate ASQ-3 subscales show that when adjusting for the other variables in the model, an increase in days of diarrhea was associated with an increased risk of being in the lower quartile in skills of fine motor and problem-solving abilities. Increasing HAZ was associated with a reduced risk of being in the lower quartile of the gross motor domain alone, while being wasted was associated with an increased risk of being in the lower quartile of communication skills. These differences may show that there are different pathways between those of diarrhea and growth and brain development, underscoring that the effect of diarrhea not only works through stunting. The independent association of diarrhea revealed in our results suggests that reducing diarrhea prevalence in children may be an important measure to enhanced neurodevelopment.

Various mechanisms may be involved in the impact of diarrhea diseases on brain development, such as for instance inflammation and/or reduced nutrient intake [22]. A possible indirect effect of childhood illnesses is the process of “functional isolation” where the child due to behavioral consequences of its condition face difficulties in eliciting appropriate caregiving behavior, and consequently fails to develop according to potential [23,24]. The hypothesis of “functional isolation” may in part explain why children burdened with pneumonia and/or diarrhea in our study have lower scores. Infected children may be weak, apathetic and irritable, and thus represent a challenge for the caregiver to provide proper responsive care.

Findings from a previous Peruvian study indicate that the various etiology of the diarrhea illness affect brain development differently, which also could explain why some studies find an association between diarrhea and neurodevelopment while other do not [4,22,25]. A limitation of our study is that the enrollment lasted for less than a year, and since it does not encompass all seasons, does not include all the variations of diarrhea illnesses. Thus, due to the lack of information on etiology in our study, our ability to demonstrate variations is limited. Developmental assessments were conducted immediately following the six months intensive follow-up, and thus a second limitation of our study, is the lack of information on long-term effects of the risk- and protective factors.

The ASQ-3 has not been formally validated for a North Indian population. To our knowledge, there are currently no up to-date-tests for this age group formally validated for this setting. However, particular for this study, we translated and adjusted the relevant ASQ-3 forms for our age groups following official recommendations. This process and its evaluation have been described elsewhere [16]. The ASQ-3 has been used previously in a clinical setting in North India [26], as well as in research in LMIC [27], and it`s sensitivity and specificity have proven to be satisfying [28]. In the present study the ASQ-3 served as a feasible tool for the purpose of collecting reliable data on developmental status in our population. Both the total and subscale scores differentiated between variables, and several variables that predicted the total ASQ-3 score confirm previous findings in this field of research [2,3]. However, since the ASQ-3 is constructed as a screening test and not a diagnostic test, it is important to underscore that diagnosis of developmental delays requires a more sophisticated confirmatory test that was not performed here.

For vulnerable children in LMIC targeted interventions to improve neurodevelopment are called for [29]. Studies have demonstrated that interventions should include both factors of responsive caregiving and learning opportunities, and nutrition for the greatest impact on early child development [21,30]. Our study provides support for these results, and furthermore, that the continuing work to reduce the burden of diarrhea illness among vulnerable children may be an important step towards enabling children to fulfill their potential. In other words, the importance of reducing the burden of illnesses may not only be important for the reduction of childhood mortality, but also to enhance quality of life through improved brain development.
